# Macroelements and Trace Elements Content in Brine-Canned Mackerel (*Scomber colias*) Subjected to High-Pressure Processing and Frozen Storage

**DOI:** 10.3390/foods9121868

**Published:** 2020-12-15

**Authors:** Ricardo Prego, Manuel Vázquez, Antonio Cobelo-García, Santiago P. Aubourg

**Affiliations:** 1 Department of Oceanography, Marine Research Institute (CSIC), 36208 Vigo, Spain; prego@iim.csic.es (R.P.); acobelo@iim.csic.es (A.C.-G.); 2 Department of Analytical Chemistry, Faculty of Veterinary Science, University of Santiago de Compostela, 27002 Lugo, Spain; manuel.vazquez@usc.es; 3 Department of Food Technology, Marine Research Institute (CSIC), 36208 Vigo, Spain

**Keywords:** chub mackerel, high-pressure processing, freezing, frozen storage, canning, macroelements, trace elements, protein denaturation, liquor loss: packaging medium

## Abstract

This study analysed the effect of prior high-pressure processing (HPP; 200–600 MPa, 2 min), freezing (−30 °C, 48 h), and frozen storage (−18 °C, 6 months) on the macroelement and trace element content in brine-canned mackerel (*Scomber colias*). Most elements (*Na*, *Ca*, *Ba*, *Mn*, *Fe*, *Cu*, *Cd*, *Sn*, *As*, *S,* and *Se*) showed an increased (*p* < 0.05) presence in mackerel muscle canned after freezing. A content increase (*p* < 0.05) was also observed for *Na* and *Sn* if prior frozen storage was also applied; on the contrary, *Ca*, *Ba*, *Mn*, *Fe*, *Cd*, *S*, and *Se* showed a content decrease (*p* < 0.05) as a result of such storage. Freezing, frozen storage, and canning led to lower values (*p* < 0.05) in canned fish for *K*, *Mg*, *Pb*, and *P*. Prior HPP led to relevant content decreases (*p* < 0.05) for *K*, *Mg*, *Ca*, *Ba*, *Mn*, *Fe*, *Pb*, and *P* contents in fish canned after the freezing step; HPP provoked additional decreases (*p* < 0.05) in *Ca*, *Ba*, and *Mn* levels in samples corresponding to 6-month frozen storage. On the contrary, prior HPP led to marked increases (*p* < 0.05) for *Cd*, *S*, and *Se* contents in all canned samples. Content changes are explained on the basis of modifications of other constituents and liquor losses from muscle.

## 1. Introduction

Marine products are known to be highly perishable. Consequently, a rapid and efficient processing and storage have to be accomplished after capture or harvest to retain the initial quality. One such process is canning. The heat treatment involved in it can substantially alter the nature of the raw starting material and provide a food product with different characteristics [1,2]. Remarkably, both enzymes and bacteria should be permanently inactivated, giving rise to a safe and durable food, provided reinfection does not occur and no negative interaction with the container is produced. Unfortunately, most marine species employed in canneries have to be stored previously. Therefore, most quality problems with canned fish can be related to the quality loss of the raw material employed [3,4].

In agreement with the short shelf life of chilled fish, freezing followed by frozen storage has been employed widely as a prior storage condition to canning process. Nevertheless, if long storage periods and/or relatively high temperatures are applied, quality assessment demonstrates that fish deterioration is produced during frozen storage since detrimental changes associated especially with lipids and proteins can occur [5,6,7]. Consequently, several complementary technologies have been applied to inhibit deterioration during fish frozen storage. Among them, high-pressure processing (HPP) has shown to retain the nutritional and sensory characteristics of seafood and lead to the inactivation of microbial growth and endogenous enzymes (hydrolytic and oxidative), so that an extended shelf life in seafood is achieved [8,9,10]. Concerning frozen seafood, HPP has shown potential industrial application as assisting freezing and thawing processing [11,12]. Furthermore, previous HPP has led to marked inhibition of damage mechanisms such as lipid hydrolysis and oxidation, and trimethylamine oxide breakdown during frozen storage of fatty and lean fish species [13,14,15]. Reports addressing the employment of HPP as assisting thermal treatment can be considered scarce [16,17]; concerning canned seafood, a previous study has recently shown a strong effect of pressure level on the subsequent development of lipid hydrolysis and oxidation of canned mackerel [18].

Marine fish live in a medium rich in mineral salts. Thus, the majority of the macroelements and trace elements considered essential for biological processes can be found in marine species [19,20,21]. However, presence of toxic trace elements has led to some health risks in commercially available fish and fish products [22,23]. Marine organisms have shown to accumulate minerals from the diet and deposit them in their skeletal tissues and organs. Furthermore, interaction of the different elements with other fish constituents has shown a great dependence on their chemical characteristics. Thus, alkali (*Na* and *K*) and alkali earth (*Mg* and *Ca*) elements have shown to be present in the cellular medium as chlorides, sulphates, or organic salts such as citrates, lactates, or pyruvates. On the contrary, transition metals (*Fe*, *Cu*, etc.) and non-positive elements (*S*, *P*, etc.) have shown to be strongly bound to other muscle constituents and give rise to a wide number of functional molecules [24,25]. Different factors such as species, tissue, maturation degree, nourishment source, season, environment, and processing may influence the concentration of elements in fish [26,27,28]. Remarkably, previous reports include abundant information on chemical changes related to constituents such as proteins and lipids during processing and storage of fish species. However, research focused on changes on mineral content during fish processing can be considered scarce.

The current research focuses on the mineral content of canned fish. In it, the effect of prior HPP (200–600 MPa, 2 min), freezing (−30 °C, 48 h), frozen storage (−18 °C, 6 months), and canning was analysed on the chemical elements content (macroelements and trace elements; essential and toxic) in brine-canned Atlantic Chub mackerel (*Scomber colias*). To the best of our knowledge, previous research related to the HPP effect on mineral content in seafood in general can be considered very scarce, and inexistent in the case of canned seafood.

## 2. Materials and Methods

### 2.1. Materials

Mackerel specimens (54 individuals; length and weight ranges: 26.3 ± 1.8 cm and 166 ± 9 g, respectively) were caught near the Galician Atlantic coast, acquired at Vigo harbour (North-Western Spain) and transported to the laboratory. Throughout this process (6–8 h), the fish were maintained in ice.

Nitric acid (Hiperpur; TMA) was obtained from PanReac AppliChem ITW Reagents (Barcelona, Spain). Element standards were obtained from Sigma-Aldrich (Steinheim, Germany). Other chemicals employed were reagent grade (Sigma-Aldrich, Madrid, Spain).

### 2.2. Initial Raw Fish, HPP, Freezing, and Frozen Storage

Six fish individuals were taken and distributed into three groups (two specimens per group). Such specimens (initial raw fish) were filleted, the white muscle being analysed independently within each group (*n* = 3).

The remaining specimens were placed in flexible polyethylene bags (8 bags; six individuals per bag), vacuum-sealed at 150 mbar (Vacuum Packaging Machine Culinary, Albipack, Águeda, Portugal) and distributed into four batches (2 bags in each batch). Bags corresponding to one of such batches were directly kept at −30 °C for 48 h (freezing treatment) and labelled as CT batch (control fish). Bags corresponding to the other three batches were subjected to HPP (200, 400, and 600 MPa for 2 min, respectively) in a 55-L high pressure unit (WAVE 6000/55 HT; NC Hiperbaric, Burgos, Spain). With this purpose, water was employed as pressurising medium at 3 MPa·s^−1^ yielding 67, 133, and 200 s as the come up times, respectively; decompression time was less than 3 s. After pressurisation, all bags were kept at −30 °C for 48 h (freezing process).

Once the freezing step was accomplished, fish belonging to one bag of each batch (CT, 200-, 400- and 600-MPa batches) were thawed (4 °C, overnight) and then employed for the canning process leading to month-0 canned fish (M-0 samples). The remaining bags (1 bag per batch) were kept at −18 °C for 6 months. At this time, fish packaged in all bags were thawed overnight at 4 °C and then employed for the canning process leading to month-6 canned fish (M-6 samples).

### 2.3. Canning and Sampling Procedure

The canning process was carried out on thawed fish corresponding to all batches. Thus, thawed fish were filleted, 45-g portions (from one fish individual and without including the tail part) being placed in flat rectangular cans (105 × 60 × 25 mm; 150 mL). Then, cans were filled with brine solution (2% *w*/*v*), vacuum-sealed and subjected to sterilisation process (125 °C, 45 min; *F*_0_ = 10 min) in a horizontal steam retort (Conservas Cerqueira, S. A., Vigo, Spain); a prior come-up time of 20 min was employed. Then, steam was cut off, the remaining steam was flushed away with air, and cans cooling was carried out at reduced pressure.

After a 3-month storage at room temperature (20 °C), the cans were opened, and the liquid phase was carefully drained off gravimetrically and filtered through a filter paper. Fish white muscle was separated, wrapped in filter paper, and used for analysis. For each batch, the fish white muscle corresponding to two cans was pooled together to carry out the chemical analyses. Three replicates (*n* = 3) of each batch were analysed.

A 3-month canned storage at room temperature was employed according to recommendation of canning manufacturers in order to obtain optimal palatability in canned products [29].

### 2.4. Moisture Determination

Moisture content of fish samples was assessed as the weight difference in homogenised muscle (1–2 g) before and after 4 h at 105 °C [30]. Results were expressed as g·kg^−1^ muscle.

### 2.5. Mineral Analysis

The content of sixteen chemical elements (*Na*, *K*, *Mg*, *Ca*, *Ba*, *Mn*, *Fe*, *Co*, *Cu*, *Cd*, *Sn*, *Pb*, *P*, *As*, *S*, and *Se*) was analysed according to the following procedure based on EPA 3050B [31]. About 1 g of ground sample was put into a digestion flask with 9 mL of 69% nitric acid (TMA) Hiperpur, 3 mL of H_2_O_2_ (for ultratrace analysis), and 3 mL of Milli-Q water. Samples, plus four blanks and four samples of certified reference material, were digested in a microwave oven (Mars-Xpress CEM Corp. Matthews, NC, USA). Samples were completely digested, and solutions were transferred to 50 mL flasks. Handling of samples was carried out inside a clean ISO 5 laminar flow cabinet (Cruma 670 FL, Barcelona, Spain). The sixteen aforementioned elements were analysed by ICP-MS (inductively coupled plasma-mass spectrometry) using an Agilent 7900 equipment (Agilent Technologies, Inc., Santa Clara, CA, USA). The quantification was carried out by external calibration with element standards traceable to NIST (National Institute of Standards and Technology) standards. The limits of detection were calculated with respect to the standard deviation of the blanks (LD = 3·SD blanks). Procedural blanks always accounted for <1% of element concentrations in the samples. Accuracy of the analytical procedures was ensured using DORM-2 certified reference material, prepared by the National Research Council of Canada (NRCC), as the quality control material (Table 1). The reference values of macroelements and *Ba* in DORM-2 were reported by Engström et al. [32]. Results were calculated as g·kg^−1^ dry muscle (macroelements) and as mg·kg^−1^ dry muscle (trace elements).

### 2.6. Statistical Analysis

Results were subjected to the ANOVA method to analyse differences resulting from the effect of the different steps carried out (HPP, freezing, frozen storage, and canning). Comparison of average values was performed using the least-squares difference (LSD) method. In all cases, analyses were carried out using the PASW Statistics 18 software for Windows (SPSS Inc., Chicago, IL, USA); differences among batches were considered significant for a confidence interval at the 95% level (*p* < 0.05).

## 3. Results

### 3.1. Changes in Alkali (Na and K) and Alkaline Earth (Mg, Ca, and Ba) Metals Content

Comparison between initial raw fish and M-0 canned samples showed an important increase (*p* < 0.05) in *Na* content resulting from the freezing step followed by the canning process (Table 2). This result could be observed both for high-pressure treated and untreated fish. Furthermore, average values of M-0 mackerel showed to be increased in all batches if a 6-month frozen storage was also applied (i.e., M-6 canned fish), such increases being significant (*p* < 0.05) for CT and 400-MPa batches. Comparison among batches led to scarce differences. Therefore, a definite trend about the effect of prior pressure level could not be concluded (*p* > 0.05).

Concerning the *K* and *Mg* levels in canned fish, HPP, freezing, and canning showed an opposite effect than in the case of Na (Table 2). Thus, a general marked decrease (*p* < 0.05) could be outlined in M-0 samples corresponding to all batches when compared with the starting raw fish; all such values were significantly decreased (*p* < 0.05) if a 6-month storage was also employed. Prior pressure applied showed a decreasing effect on both elements content for M-0 mackerel. Hence, lower values (*p* < 0.05) were observed in canned fish corresponding to 400- and 600-MPa batches. However, significant differences (*p* > 0.05) were not detected among canned samples if a 6-month storage was taken into account, although fish corresponding to the control batch revealed the highest average values for both essential elements.

**Figure 1:**
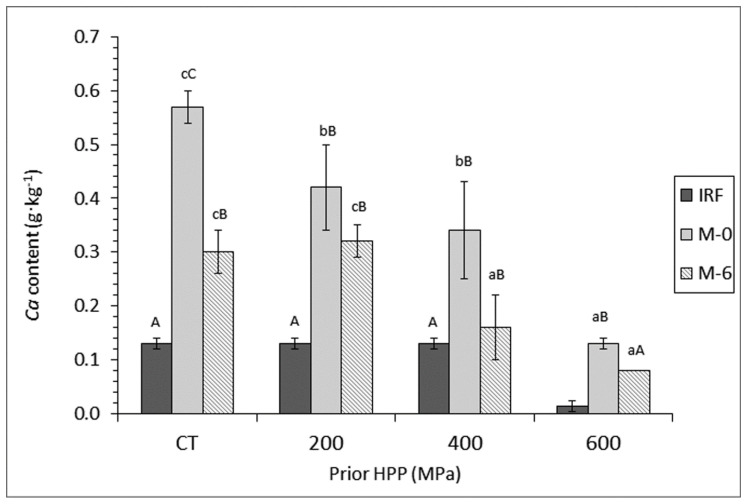
Determination of *Ca* content (g·kg^−1^ dry muscle) * in initial raw fish (IRF) and canned mackerel subjected to high-pressure processing (HPP), freezing, and frozen storage **. * Mean values of three replicates (*n* = 3). Standard deviations are indicated by bars. For each frozen storage time (0 or 6 months), different lower-case letters (a–c) denote significant differences (*p* < 0.05) as a result of HPP. For each HPP condition, capital letters (A–C) denote significant differences (*p* < 0.05) as a result of frozen storage time. ** Sample name abbreviations: CT (control batch; without HPP), M-0 (fish canned after freezing) and M-6 (fish canned after a 6-month frozen storage).

Evolution of *Ca* content (Figure 1) provided a similar response than that of *Ba* (Table 2) to the different processing steps included in the current study. Thus, comparison between initial raw fish and CT canned fish corresponding to month-0 storage showed a marked increase (*p* < 0.05) as a result of HPP, freezing, and canning. However, content on both elements provided a significant decrease (*p* < 0.05) in CT canned samples as a result of including a 6-month frozen storage period. Remarkably, *Ca* average levels in M-6 fish were higher than in initial raw fish (except for 600-MPa batch), while levels of *Ba* in canned samples were lower (*p* < 0.05) than in starting fish for all batches. On the other side, a marked effect can be signalled for prior pressure on the content of both elements. Thus, a significant decrease (*p* < 0.05) was implied by increasing the prior pressure applied in samples corresponding to M-0 and M-6 conditions. For both elements, canned fish corresponding to 600-MPa batch showed lower values (*p* < 0.05) than their counterpart samples corresponding to the other three batches.

### 3.2. Changes in Transition Metals Content (Mn, Fe, Co, Cu, and Cd)

**Figure 2:**
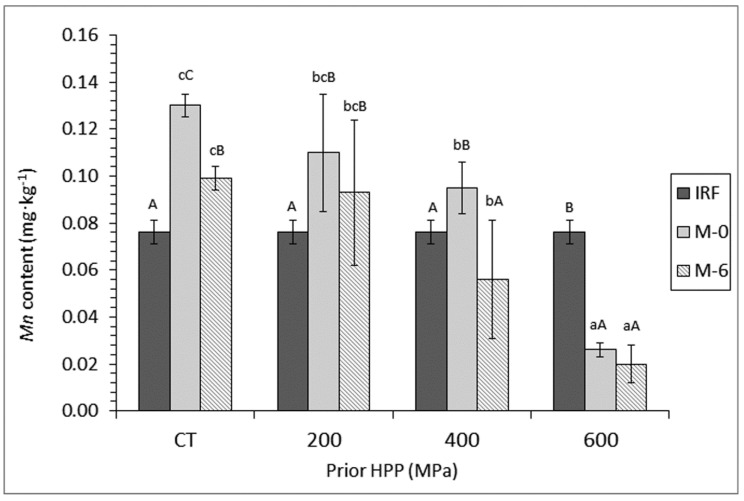
Determination of *Mn* content (mg·kg^−1^ dry muscle) * in initial raw fish (IRF) and canned mackerel subjected to high-pressure processing (HPP), freezing, and frozen storage **. * Mean values of three replicates (*n* = 3). Standard deviations are indicated by bars. For each frozen storage time (0 or 6 months), different lower-case letters (a–d) denote significant differences (*p* < 0.05) as a result of HPP. For each HPP condition, capital letters (A–C) denote significant differences (*p* < 0.05) as a result of frozen storage time. ** Sample name abbreviations as expressed in Figure 1.

In the case of *Mn*, comparison between initial raw fish and canned fish corresponding to month-0 frozen storage showed a significant increase (*p* < 0.05), except for canned muscle previously submitted to a 600-MPa treatment that showed a significant decrease (*p* < 0.05) (Figure 2). Furthermore, comparison between average values of M-0 and M-6 canned samples provided a decreasing effect of the prior frozen storage period. This effect was significant (*p* < 0.05) in the case of CT and 400-MPa batches. A strong decreasing effect (*p* < 0.05) on the content of this essential element could be accorded to prior pressure processing. Thus, for both M-0 and M-6 canned samples, a progressive decrease (*p* < 0.05) by increasing the pressure level was proved.

Levels of *Fe* in canned fish showed a great general increase (*p* < 0.05) by comparing M-0 samples and initial raw fish (Table 3). However, a definite effect could not be implied (*p* < 0.05) when a subsequent frozen storage was encountered. Some differences were detected resulting from the prior HPP, although a definite trend could not be concluded (*p* > 0.05) for the presence of this essential element.

The levels of *Co* and *Cu* in canned fish showed a general increase (*p* < 0.05) after HPP, freezing and canning (Table 3). However, the employment of a subsequent 6-month frozen storage led to scarce differences when compared with M-0 samples; thus, a remarkable content decrease (*p* < 0.05) was obtained in M-6 canned fish if previously subjected to a 600-MPa treatment. Concerning the effect of prior pressure treatment, a definite trend could not be implied in canned samples for both essential elements. For *Co*, higher average values were obtained in the 600-MPa batch when comparing the different kinds of batches corresponding to M-0 canned fish. Meantime, CT batch provided the highest average values if a prior 6-month frozen storage was also accomplished. In the case of *Cu*, higher average values were obtained in 400- and 600-MPa batches at the M-0 condition; for M-6 fish, *Cu* levels of 400-MPa batch were found higher (*p* < 0.05) than those corresponding to its counterpart batches.

**Figure 3:**
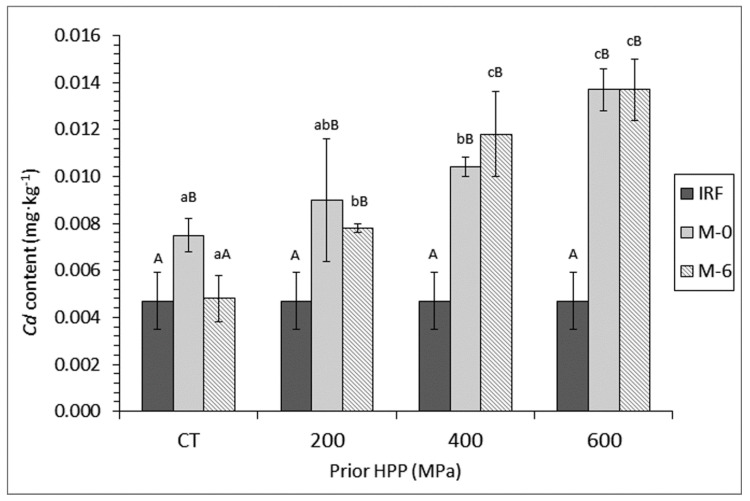
Determination of *Cd* content (mg·kg^−1^ dry muscle) * in initial raw fish (IRF) and canned mackerel subjected to high-pressure processing (HPP), freezing, and frozen storage **. * Mean values of three replicates (*n* = 3). Standard deviations are indicated by bars. For each frozen storage time (0 or 6 months), different lower-case letters (a–c) denote significant differences (*p* < 0.05) as a result of HPP. For each HPP condition, capital letters (A, B) denote significant differences (*p* < 0.05) as a result of frozen storage time. ** Sample name abbreviations as expressed in Figure 1.

Determination of *Cd* content in canned fish showed a general increase (*p* < 0.05) with processing when comparing the initial raw fish and samples corresponding to month-0 condition (Figure 3). However, comparison between M-0 and M-6 canned fish from the CT batch provided a marked *Cd* content decrease (*p* < 0.05) as a result of subjecting the fish to a frozen storage period. On the contrary, canned fish previously subjected to high-pressure did not provide differences (*p* > 0.05) as a result of the frozen storage period. Interestingly, a strong effect of prior pressure level applied was observed (*p* < 0.05). Thus, increasing contents for this toxic element were obtained with pressure level in samples corresponding to prior 0 and 6 months of frozen storage. Remarkably, higher *Cd* values were observed in 600- and 400-MPa batches when compared with their counterpart controls.

### 3.3. Changes in the Content of Other Metals (Sn and Pb)

A general increase (*p* < 0.05) in *Sn* content was proved in canned samples corresponding to month-0 condition when compared with initial fish values (Table 4). If a frozen storage period is included as prior treatment to canning, an additional increase (*p* < 0.05) in *Sn* average levels in canned fish was observed in all batches. Differences were significant (*p* < 0.05) only for the CT batch. No significant effect (*p* > 0.05) of prior HPP was implied on the content of this toxic metal in canned fish; however, higher average values were observed in 200- and 400-MPa batches.

Determination of *Pb* content in canned fish revealed a strong general decrease (*p* < 0.05) as a result of HPP, freezing, and canning (Table 4). On the contrary, if a prior 6-month frozen storage is employed, higher average values were obtained in all batches if compared with canned samples corresponding to month-0 storage. However, such M-6 canned values were substantially lower (*p* < 0.05) than those corresponding to the initial fish. A decreasing effect of prior pressure-level applied could be outlined in M-0 samples, the lowest average values for this toxic element being obtained in the 600-MPa batch.

### 3.4. Changes in Metalloids (As and Se) Content

Average levels of *As* increased with HPP, freezing and canning in all kinds of samples; differences were found significant (*p* < 0.05) in the CT and 200-MPa batches (Table 4). However, if a 6-month frozen storage period is also included as pre-treatment, comparison with month-0 samples led to lower average values, but differences were not significant (*p* > 0.05). Concerning the effect of HPP, scarce differences were detected and a definite trend could not be signalled (*p* > 0.05) in values corresponding to this toxic element.

In all kinds of canned fish, higher *Se* levels were detected than in initial raw fish (Table 4). Nevertheless, comparison between M-0 and M-6 canned samples showed a relevant decrease as a result of including a 6-month frozen storage period; remarkably, this decrease was significant (*p* < 0.05) in CT, 400-, and 600-MPa batches. An average *Se* value increase was observed in canned fish by increasing the pressure level applied. Thus, lower values (*p* < 0.05) for this essential element were obtained in CT batch when compared with 400- and 600-MPa fish (M-0 samples) and when compared with all high-pressure treated batches (M-6 samples).

### 3.5. Changes in Non-Metal Elements (P and S) Content

A general decrease (*p* < 0.05) of *P* content was detected in canned samples corresponding to month-0 condition when compared with initial fish values (Table 5). Furthermore, subjecting the mackerel to a prior frozen storage period of 6 months led the *P* level in all batches to lower values (*p* < 0.05) than those obtained for M-0 fish. A decreasing effect with pressure level could also be outlined for the content of this macroelement; this effect was more remarkable in samples corresponding to M-0 condition. Thus, a lower (*p* < 0.05) value in month-0 canned fish corresponding to 600-MPa treatment was obtained when compared with their counterparts from CT and 200-MPa batches. For M-6 samples, canned fish related to 400- and 600-MPa batches revealed lower average values than their counterparts from CT and 200-MPa batches.

Related to *S* levels, average contents showed a general increase with processing when comparing initial values corresponding to raw fish and month-0 canned fish. Differences were significant (*p* < 0.05) in all batches except for 200-MPa fish (Table 5). Additionally, comparison between M-0 and M-6 canned samples provided a general decrease of average levels of this macroelement resulting from the frozen storage period. Differences were significant (*p* < 0.05) for the CT batch. Concerning the effect of prior pressure applied, a higher (*p* < 0.05) *S* content was detected in canned fish belonging to the highest pressure applied for both month-0 and month-6 conditions.

### 3.6. Changes of Moisture Content

Comparison between initial raw fish and canned fish showed no effect (*p* > 0.05) of HPP (200 and 400 MPa conditions), freezing, frozen storage, and canning on the moisture content (Table 6). However, if canned fish corresponding to the highest pressure condition is considered, a marked decrease of the moisture average value was obtained in canned fish, differences being significant (*p* < 0.05) if a 6-month storage period is also encountered as prior treatment. Furthermore, comparison among batches showed the lowest average values in fish corresponding to 600-MPa treatment both for M-0 and M-6 samples; differences with control were significant (*p* < 0.05) in fish canned after the freezing step and after a 6-month frozen storage.

## 4. Discussion

### 4.1. Changes in Moisture Content in Canned Fish

Differences between moisture values corresponding to raw fish and canned fish (Table 6) can be explained as a result of the different processing steps carried out. Thus, two basic and opposite effects can be pointed out. On one side, denaturation of muscle proteins during the different steps of processing (pressurisation, freezing, frozen storage, and canning), especially during the sterilisation step, would lead to a decrease of water-holding capacity, so that a substantial loss of water from the fish muscle should be produced [3,7,10]. On the other side, an interaction between the fish muscle and the hydrophilic brine-packaging medium ought to be produced [33,34]. Therefore, the fish muscle would be imbibed in the liquid medium, thus leading to a moisture content increase in canned muscle. Current results obtained indicate that most canned fish (CT, 200-, and 400-MPa batches) has not shown a significant effect (*p* > 0.05) on moisture level. Consequently, an equilibrium between both effects can be implied in such cases. On the contrary, samples corresponding to the highest pressure level batch revealed a marked decrease (*p* < 0.05) of moisture content, this showing an important effect of prior HPP on moisture content decrease in canned fish muscle.

Previous studies have shown that no changes in moisture content have been observed in canned fish muscle when packaged under an aqueous medium (i.e., brine) [34]. However, if an oily packaging medium was employed such as soya bean oil [35] or olive oil [34], a relevant decrease was detected in canned fish muscle. In a comparative study, brine and olive oil were used as packaging methods for canned freshwater nase (*Chondrostoma nasus*) [33]; it could be observed that both oil- and brine-packaged fish lost moisture, but this loss was higher in oil-canned fish as well as by increasing the *Fo* value applied.

### 4.2. Changes in Mineral Content in Canned Fish

Changes in mineral content (i.e., dry muscle basis) between the initial raw fish and canned samples have shown different trends (increase, decrease, or no variation) according to the element taken into account and the processing steps considered. In order to justify variation of mineral contents in the final canned fish, some revision of the most important events produced in each single processing step ought to be carried out.

Thus, HPP application on marine food has been focused on its ability for protein denaturation and consequently, the inhibition of endogenous and microbial deteriorative enzymes. The intensity and reversibility of this effect has shown to be strongly dependent on the strength of HPP conditions [9,10,13]. As a result of marked changes in protein structure by HPP, a water-holding capacity decrease is reported to occur, so that the resulting liquor loss may have an important detrimental effect on mineral retention in the fish muscle. According to data presented in Table 6, this effect would be expected to be especially important in the 600-MPa canned batch. On the other side, proteins in general, but the sarcoplasmic fraction especially (around 25–30% of total proteins in fish), have shown to be especially labile to HPP, so that marked breakdown and content decrease have been proved in fatty [36] and lean [37] fish. As a consequence of this protein loss in the fish muscle, a relative content increase on other muscle constituents, such as minerals, would be expected to occur. Unfortunately, previous research related to the effect of HPP on mineral content in canned seafood and processed seafood in general is, to the best of our knowledge, not yet available.

Concerning the freezing and frozen storage of fish, different mechanisms of damage have been pointed out as being responsible for nutritional and sensory losses [5,7]. Among them, protein denaturation, microstructural changes, and lipid oxidation development have been signalled as most important for quality retention. As a result of freezing and frozen storage, the muscle becomes harder, more fibrous, less elastic, and losses its water-holding capacity [6,38]. As expressed for HPP, the resulting loss of water-holding capacity of proteins can be especially important for mineral content, since liquor produced from the muscle, especially during the required thawing step, can lead to important losses in mineral content. This mineral release would be expected to increase with time and temperature of frozen storage. However, previous research related to this loss can be considered scarce. Remarkably, one especially important consequence for quality retention would be the increase of the non-*Fe* content due to release of *Fe* from heme-*Fe* complexes by oxidative cleavage of the porphyrin ring [39,40].

Related to the effect of the canning process (namely, sterilisation step), and thermal treatments in general, marine species constituents have been revealed to be highly sensitive [2,41]. Among the most important events, heat breakdown and oxidation of constituents (namely, protein and lipid fractions), leaching of water-soluble constituents, and toughening and drying of fish muscle can be mentioned, all of them susceptible to exert an effect on mineral content [42,43]. Related to mineral content, and as for HPP and frozen storage, liquor losses from canned fish muscle may have a detrimental effect on mineral content, thus leading to substantial content decreases. Consequently, reduction in mineral contents of fish during the heating process may be related to the protein denaturation and release of these elements with the loss of water as free salts, possibly associated with soluble free amino acids, hydrophobic vitamins, and uncoagulated proteins [44,45,46]. This loss can be especially important when an aqueous medium is employed as a packaging medium. Remarkably, a higher fat content in the fish flesh produces lower losses of minerals, thus indicating a kind of interaction between both kinds of constituents [47,48]. On the other side, it is known that denatured proteins become more reactive and can be damaged easily by interacting with other constituents, especially if a strong process such as sterilisation is concerned. Furthermore, and as expressed above for frozen storage, release of prooxidant elements such as non-heme *Fe* from heme-*Fe* complexes may have important consequences on rancidity stability of fish muscle [39,49]. Thus, canned fish may undergo a substantial lipid oxidation development, thus leading to lipid breakdown and production of low-molecular-weight molecules susceptible to be lost from the canned fish muscle [3,34]. Therefore, a content decrease in constituents such as proteins and lipids in the canned fish muscle would lead to a relative increase of other constituents such as minerals.

Previous research has addressed the effect of canning on mineral content in fish muscle. Thus, some loss in minerals (*Na*, *K*, *Mg*, *P*, *Cu*, *Fe*, and *Ca*) from the muscle into the packaging medium was detected by Seet and Brown [50] in water-packaged canned tuna (*Thunnus alalunga*). Later on, Castrillón et al. [35] obtained a water and protein loss and fat content increase after albacore (*Thunnus alalunga*) canning (sterilisation at 115 °C for 55 or 90 min) by using soy bean oil as packaging medium; from raw to steamed fish, some elements content decreased (*Mg*, *K*, and *P*) and others remained the same (*Ca*, *Na*, *Zn*, *Cu*, and *Fe*). Ganjavi et al. [45] showed that defrosting, cooking, and sterilisation reduced the contents of *Pb* and *Cd* considerably in oil-canned yellowfin tuna (*Thunnus albacares*) and skipjack tuna (*Katsuwonus pelamis*) from the Persian Gulf and Oman Sea.

Consequently, and taking into account the different effects that each single processing step may have on the current canned mackerel, two opposite effects can be signalled related to changes in the mineral content in the final canned muscle. One side, each of the different processing steps (HPP, freezing, frozen storage, and canning), especially sterilisation, would lead to denaturalisation, oxidation breakdown, and partial loss of the main constituents (proteins and lipids, especially) [7,10,41]. Therefore, a relative increase of other constituent content such as macrominerals and trace elements would be expected to occur. On the other side, modifications of the different constituents and consequently, breakdown of binding of minerals to other constituents, as well as liquor losses from the muscle would lead to a partial loss of minerals into the packaging medium. Among the different constituent modifications, protein denaturation can be of special significance for mineral content in fish muscle, as leading to a decreased water-holding capacity [10,45], and resulting in an increasing liquor loss susceptible to increase the mineral loss. The importance of this effect would depend on the kind of binding to other constituents of the fish muscle and the more or less hydrophilic/lipophilic nature of molecules they are integrated in [24,25]. Thus, elements whose linkage to other constituents is easily lost during any of the processing steps would lead to a decreased content. On the contrary, those whose linkage to other constituents is not modified during processing would not be likely to be lost and would increase their relative content in canned fish muscle according to the content decrease of other constituents such as proteins and lipids.

Consequently, mineral contents measured in the present study can be considered the result of these two opposite effects. Those elements that have shown to increase their content would imply a predominance of the first factor mentioned (i.e., partial loss of other constituents). On the contrary, a content decrease would signify that the second factor (i.e., liquor losses) has been more important. Furthermore, a balanced incidence of both factors would be produced when no substantial differences are found.

## 5. Conclusions

The content on different macroelements and trace elements in canned Atlantic Chub mackerel was analysed, taking into account the effect that prior HPP, freezing, frozen storage, and canning may exert. As a result, different trends were implied on the basis of changes in other constituents of the muscle (namely, protein denaturation, water loss, and lipid oxidation), liquor losses from the muscle, interaction of the fish muscle with the hydrophilic brine-packaging medium, the kind of binding of minerals to other constituents of the fish muscle and the more or less hydrophilic/lipophilic behaviour of molecules they are integrated in. It is concluded that elements that have shown to increase their content in canned muscle would imply a predominance of a partial loss of other constituents (i.e., protein and lipids fractions). On the contrary, a content decrease would signify that mineral losses from the muscle into liquor losses from the canned muscle resulting from protein denaturation would be more important. Furthermore, a balanced incidence of opposite factors would be produced when no substantial differences are found.

Thus, most elements (*Na*, *Ca*, *Ba*, *Mn*, *Fe*, *Cu*, *Cd*, *Sn*, *As*, *S*, and *Se*) showed an increased (*p* < 0.05) presence in mackerel muscle canned after freezing (M-0 samples). An additional content increase (*p* < 0.05) was observed by means of subjecting the mackerel muscle to a prior 6-month frozen storage for *Na* and *Sn* in canned fish, while other elements (*Ca*, *Ba*, *Mn*, *Fe*, *Cd*, *S*, and *Se*) showed decreased levels (*p* < 0.05) as a result of subjecting the fish to such a frozen storage period. On the contrary, prior freezing and canning led to lower values (*p* < 0.05) in canned fish for *K*, *Mg*, *Pb*, and *P*, such values being lowered (*p* < 0.05) if a prior 6-month frozen storage was also employed.

Concerning the effect of prior HPP, a relevant content decrease (*p* < 0.05) could be observed for *K*, *Mg*, *Ca*, *Ba*, *Mn*, *Fe*, *Pb*, and *P* in fish canned after the freezing step (namely, M-0 samples); additionally, a decrease (*p* < 0.05) was implied in *Ca*, *Ba*, and *Mn* values if a 6-month frozen storage period was undertaken (i.e., M-6 samples). On the contrary, prior HPP led to a marked increase (*p* < 0.05) with pressure level applied for *Cd*, *S*, and *Se* contents both in M-0 and M-6 canned samples. Finally, no effect (*p* > 0.05) of prior HPP was implied in canned mackerel for *Na*, *Sn*, and *As* levels. In most cases, HPP effect increased with pressure level.

Previous reports on mineral content in processed seafood can be considered scarce in general, being negligible in the case of high-pressure treated seafood. The current study represents a first approach on the knowledge of the effect of different pre-treatments on the content of macroelements and trace elements presence in canned fish. Further research ought to be addressed on the knowledge of the different biomolecules minerals are integrated on in order to justify the content increase/decrease resulting from processing. Additionally, and on the basis of the great impact of essential and toxic element levels in canned fish, further research ought to be addressed to analyse the effect of different pre-processing conditions such as prior frozen storage time, packaging medium employed for canning, and sterilisation condition (time and temperature).

## Figures and Tables

**Table 1:** Accuracy control of the analytical procedures for the assessment of macroelements and trace elements *.

Element	Certified	Measured	Unit
**MACROELEMENTS**			
*Ca*	0.62 ± 0.05	0.62 ± 0.09	g·kg^−1^
*K*	18.9 ± 1.1	17.0 ± 0.5	“
*Mg*	1.05 ± 0.05	1.15 ± 0.05	“
*Na*	5.06 ± 0.07	5.72 ± 0.17	“
*P*	9.9 ± 0.1	10.6 ± 0.5	“
*S*	8.9 ± 0.5	8.5 ± 0.2	“
**TRACE ELEMENTS**			
*As*	18.0 ± 1.1	17.3 ± 1.8	mg·kg^−1^
*Ba*	2.34 ± 0.03	2.4 ± 0.3	“
*Cd*	0.043 ± 0.008	0.038 ± 0.002	“
*Co*	0.182 ± 0.031	0.16 ± 0.02	“
*Cu*	2.34 ± 0.16	1.92 ± 0.23	“
*Fe*	142 ± 10	105 ± 15	“
*Pb*	0.065 ± 0.007	0.047 ± 0.007	“
*Mn*	3.66 ± 0.34	3.02 ± 0.29	“
*Se*	1.40 ± 0.09	1.41 ± 0.12	“
*Sn*	0.023 ± 0.001	0.026 ± 0.009	“

* Data expressed as mean value ± standard deviation (*n* = 4). Certified reference material was DORM-2 (National Research Council of Canada (NRCC)), where macroelements and *Ba* values were reported by Engström et al. [32].

**Table 2:** Determination of different alkali (*Na* and *K*) and alkaline earth (*Mg* and *Ba*) metal elements contents * in initial raw fish and canned mackerel subjected to different processing conditions **.

Element	Frozen Storage Time (Months)	High-Pressure Processing (HPP) (MPa)			
		CT	200	400	600
*Na* (g·kg^−1^ dry muscle)	Initial raw fish	2.31 A (0.04)	2.31 A (0.04)	2.31 A (0.04)	2.31 A (0.04)
	0	5.07 abB (0.10)	5.14 bB (0.06)	4.95 aB (0.10)	4.98 abB (0.28)
	6	5.48 aC (0.22)	5.46 aB (0.26)	5.63 aC (0.24)	5.40 aB (0.19)
*K* (g·kg^−1^ dry muscle)	Initial raw fish	3.46 C (0.19)	3.46 C (0.19)	3.46 C (0.19)	3.46 C (0.19)
	0	1.53 bB (0.05)	1.55 bB (0.12)	1.37 aB (0.02)	1.29 aB (0.10)
	6	1.10 aA (0.13)	1.05 aA (0.11)	1.05 aA (0.03)	1.00 aA (0.05)
*Mg* (g·kg^−1^ dry muscle)	Initial raw fish	0.51 C (0.02)	0.51 C (0.02)	0.51 C (0.02)	0.51 C (0.02)
	0	0.18 bB (0.01)	0.19 bB (0.01)	0.16 aB (0.00)	0.15 aB (0.01)
	6	0.14 aA (0.01)	0.13 aA (0.00)	0.13 aA (0.00)	0.13 aA (0.01)
*Ba* (mg·kg^−1^ dry muscle)	Initial raw fish	0.054 B (0.003)	0.054 B (0.003)	0.054 B (0.003)	0.054 B (0.003)
	0	0.085 cC (0.03)	0.058 bB (0.06)	0.047 bB (0.007)	0.017 aA (0.004)
	6	0.042 cA (0.007)	0.039 cA (0.003)	0.026 bA (0.005)	0.015 aA (0.002)

* Mean values of three replicates (*n* = 3). Standard deviations are indicated in brackets. ** In each row, different lower-case letters (a–c) denote significant differences (*p* < 0.05) as a result of HPP. In each column, capital letters (A–C) denote significant differences (*p* < 0.05) as a result of freezing and frozen storage.

**Table 3:** Determination (mg·kg^−1^ dry muscle) of different transition metal elements (*Fe*, *Co*, and *Cu*) contents * in initial raw fish and canned mackerel subjected to different processing conditions **.

Element	Frozen Storage Time (Months)	High-Pressure Processing (HPP) (MPa)			
		CT	200	400	600
*Fe*	Initial raw fish	3.35 A (0.01)	3.35 A (0.01)	3.35 A (0.01)	3.35 A (0.01)
	0	7.51 cC (0.05)	6.64 bB (0.45)	7.18 bcB (0.35)	5.89 aB (0.11)
	6	6.48 aB (0.07)	7.28 bC (0.04)	6.51 abB (0.59)	6.86 abC (0.41)
*Co*	Initial raw fish	0.0018 A (0.0003)	0.0018 A (0.0003)	0.0018 A (0.0003)	0.0018 A (0.0003)
	0	0.0049 abB (0.0004)	0.0055 bcC (0.0007)	0.0042 aB (0.0004)	0.0065 cC (0.0006)
	6	0.0051 aB (0.0012)	0.0041 aB (0.0003)	0.0042 aB (0.0002)	0.0044 aB (0.0007)
*Cu*	Initial raw fish	0.36 A (0.03)	0.36 A (0.03)	0.36 A (0.03)	0.36 A (0.03)
	0	0.54 abB (0.05)	0.51 aB (0.04)	0.71 cB (0.07)	0.66 bcC (0.08)
	6	0.59 aB (0.06)	0.58 aB (0.05)	0.70 bB (0.02)	0.49 aB (0.05)

* Mean values of three replicates (*n* = 3). Standard deviations are indicated in brackets. ** In each row, different lower-case letters (a–c) denote significant differences (*p* < 0.05) as a result of HPP. In each column, capital letters (A–C) denote significant differences (*p* < 0.05) as a result of freezing and frozen storage.

**Table 4:** Determination (mg·kg^−1^ dry muscle) of two metals (*Sn* and *Pb*) and two metalloid (*As* and *Se*) contents * in initial raw fish and canned mackerel subjected to different processing conditions **.

Element	Frozen Storage Time (Months)	High-Pressure Processing (HPP) (MPa)			
		CT	200	400	600
*Sn*	Initial raw fish	0.0011 A (0.0006)	0.0011 A (0.0006)	0.0011 A (0.0006)	0.0011 A (0.0006)
	0	0.0032 aB (0.0004)	0.0042 aB (0.0005)	0.0042 aB (0.0009)	0.0034 aB (0.0008)
	6	0.0045 aC (0.0002)	0.0048 aB (0.0003)	0.0053 aB (0.0005)	0.0047 aB (0.0005)
*Pb*	Initial raw fish	0.0127 B (0.0037)	0.0127 B (0.0037)	0.0127 B (0.0037)	0.0127 B (0.0037)
	0	0.0032 abA (0.0012)	0.0032 bA (0.0009)	0.0025 bA (0.0001)	0.0015 aA (0.0006)
	6	0.0040 aA (0.0013)	0.0037 aA (0.0000)	0.0039 aA (0.0015)	0.0030 aA (0.0008)
*As*	Initial raw fish	1.02 A (0.05)	1.02 A (0.05)	1.02 A (0.05)	1.02 A (0.05)
	0	1.20 aB (0.01)	1.47 bB (0.09)	1.30 abA (0.30)	1.16 aA (0.08)
	6	1.12 aAB (0.16)	1.15 aAB (0.09)	1.13 aA (0.10)	1.17 aA (0.10)
*Se*	Initial raw fish	0.42 A (0.02)	0.42 A (0.02)	0.42 A (0.02)	0.42 A (0.02)
	0	0.81 aC (0.07)	0.77 aB (0.06)	0.92 bC (0.01)	1.03 cC (0.04)
	6	0.62 aB (0.03)	0.74 bB (0.03)	0.76 bB (0.03)	0.78 bB (0.02)

* Mean values of three replicates (*n* = 3). Standard deviations are indicated in brackets. ** In each row, different lower-case letters (a–c) denote significant differences (*p* < 0.05) as a result of HPP. In each column, capital letters (A–C) denote significant differences (*p* < 0.05) as a result of freezing and frozen storage.

**Table 5:** Determination (mg·kg^−1^ dry muscle) of different non-metal elements (*P* and *S*) contents * in initial raw fish and canned mackerel subjected to different processing conditions **.

Element	Frozen Storage Time (Months)	High-Pressure Processing (HPP) (MPa)			
		CT	200	400	600
*P*	Initial raw fish	2.61 C (0.05)	2.61 C (0.05)	2.61 C (0.05)	2.61 C (0.05)
	0	1.95 cB (0.05)	1.86 bcB (0.15)	1.67 abB (0.13)	1.55 aB (0.03)
	6	1.32 aA (0.07)	1.35 aA (0.10)	1.26 aA (0.08)	1.23 aA (0.09)
*S*	Initial raw fish	2.72 A (0.06)	2.72 A (0.06)	2.72 A (0.06)	2.72 A (0.06)
	0	2.87 aB (0.06)	2.74 aA (0.11)	2.90 aB (0.07)	3.13 bB (0.09)
	6	2.74 aA (0.02)	2.68 aA (0.09)	2.73 aAB (0.10)	3.11 bB (0.06)

* Mean values of three replicates (*n* = 3). Standard deviations are indicated in brackets. ** In each row, different lower-case letters (a–c) denote significant differences (*p* < 0.05) as a result of the HPP. In each column, capital letters (A–C) denote significant differences (*p* < 0.05) as a result of freezing and frozen storage.

**Table 6:** Moisture content (g·kg^−1^ muscle) * of initial raw fish and canned mackerel subjected to different processing conditions **.

Prior Frozen Storage Time (Months)	High-Pressure Processing (HPP) (MPa)			
	CT	200	400	600
Initial raw fish	717.2 AB (19.3)	717.2 A (19.3)	717.2 A (19.3)	717.2 B (19.3)
0	725.7 bB (4.1)	730.6 bA (19.2)	709.4 abA (18.5)	679.4 aAB (28.8)
6	707.8 bA (3.1)	699.3 abA (12.9)	709.1 bA (11.9)	678.4 aA (12.1)

* Mean values of three replicates (*n* = 3). Standard deviations are indicated in brackets. ** In each row, different lower-case letters (a, b) denote significant differences (*p* < 0.05) as a result of HPP. In each column, capital letters (A, B) denote significant differences (*p* < 0.05) as a result of freezing and frozen storage time.
